# 4-hydroxy-3-methoxycinnamic acid regulates orexigenic peptides and hepatic glucose homeostasis through phosphorylation of FoxO1

**DOI:** 10.1038/emm.2017.253

**Published:** 2018-02-02

**Authors:** Ann W Kinyua, Chang Mann Ko, Khanh V Doan, Dong Joo Yang, My Khanh Q Huynh, Sang Hyun Moh, Yun-Hee Choi, Ki Woo Kim

**Affiliations:** 1 Departments of Pharmacology and Global Medical Science, Wonju College of Medicine, Yonsei University, Wonju, Republic of Korea; 2 Anti-aging Research Institute of BIO-FD&C Co. Ltd., Incheon, Republic of Korea

## Abstract

4-hydroxy-3-methoxycinnamic acid (ferulic acid, FA) is known to have numerous beneficial health effects, including anti-obesity and anti-hyperglycemic properties. However, the molecular networks that modulate the beneficial FA-induced metabolic effects have not been well elucidated. In this study, we explored the molecular mechanisms mediating the beneficial metabolic effects of FA. In mice, FA protected against high-fat diet-induced weight gain, reduced food intake and exhibited an overall improved metabolic phenotype. The food intake suppression by FA was accompanied by a specific reduction in hypothalamic orexigenic neuropeptides, including agouti-related protein and neuropeptide Y, with no significant changes in the anorexigenic peptides pro-opiomelanocortin and cocaine and amphetamine-regulated transcript. FA treatment also inhibited fat accumulation in the liver and white adipose tissue and suppressed the expression of gluconeogenic genes, including phosphoenolpyruvate carboxylase and glucose-6-phosphatase. Furthermore, we show that FA phosphorylated and inactivated the transcription factor FoxO1, which positively regulates the expression of gluconeogenic and orexigenic genes, providing evidence that FA might exert its beneficial metabolic effects through inhibition of FoxO1 function in the periphery and the hypothalamus.

## Introduction

Obesity is a chronic disorder of energy homeostasis that is associated with numerous health complications, including type II diabetes.^1^ Dysregulation of whole body energy metabolism resulting from an imbalance in energy consumption and energy expenditure is the primary cause of obesity development.^2^ The central nervous system (CNS), particularly the hypothalamus, has an important role in maintaining whole-body energy homeostasis by integrating nutritional signals from peripheral organs and therefore regulates feeding and energy expenditure.^3, 4^ The arcuate nucleus of the hypothalamus consists of two distinct neuronal populations that regulate not only food intake but also energy expenditure and body weight. The orexigenic agouti-related protein (AgRP)- and neuropeptide Y (NPY)-expressing neurons stimulate feeding, whereas the anorexigenic pro-opiomelanocortin (POMC)- and cocaine and amphetamine-regulated transcript (CART)-expressing neurons inhibit food intake and increase energy expenditure.^5, 6, 7, 8, 9^

The transcription factor FoxO1 is a downstream target of the PI3K/AKT pathway, which is important for various physiological processes including the regulation of energy homeostasis.^10, 11, 12^ In the hypothalamus, insulin and leptin converge at the AKT/FoxO1 pathway to regulate the expression of both orexigenic and anorexigenic genes.^13, 14, 15, 16^ FoxO1 upregulates the expression of AgRP but inhibits POMC expression, therefore promoting food intake. However, leptin and insulin antagonize the effects of FoxO1 by phosphorylating and inactivating FoxO1 to suppress food intake.^17, 18, 19^ In peripheral tissues, FoxO1 regulates the expression of key gluconeogenic genes, phosphoenolpyruvate carboxylase (PEPCK) and glucose-6-phosphatase (G6P),^20^ demonstrating the important role of FoxO1 in the regulation of glucose homeostasis.

4-hydroxy-3-methoxycinnamic acid (ferulic acid, FA) is found in grains, fruits and even in marine algae.^21, 22^ Previous studies have demonstrated the various beneficial metabolic effects of FA, including reduced serum and hepatic lipid accumulation in diabetic rats, protection against lipid induced insulin resistance^23, 24^ and improved glucose tolerance and insulin sensitivity.^25, 26, 27^ Whereas most studies on the anti-diabetic and anti-hyperglycemic activities of FA have focused on peripheral tissues, the molecular networks mediating these effects have not been clearly elucidated. Moreover, the CNS-mediated metabolic roles of FA have yet to be explored.

In this study, we observed that FA has a protective role against the development of high-fat diet (HFD)-induced obesity by reducing food intake, and improving glucose metabolism and insulin sensitivity. FA also suppressed lipid accumulation in the liver and white adipose tissue. The beneficial effects of FA were accompanied by the inhibition of gluconeogenic genes, PEPCK and G6P, in the liver and the suppression of orexigenic genes, AgRP and NPY, in the hypothalamus. Interestingly, we found that FA phosphorylated and led to the nuclear exclusion of FoxO1, a known transcription factor that is an important regulator of the expression of gluconeogenic and orexigenic genes. Therefore, our data suggest that the beneficial metabolic effects of FA might be a result of the combined effects of FA on the inhibition of hepatic glucose production and the suppression of food intake and that these effects may be exerted through suppression of FoxO1 activity.

## Materials and methods

### Animal experiments

All animal experiments were approved by the Institutional Animal Care and Use Committee of Yonsei University, Wonju College of Medicine. C57BL/6 male mice were housed in a controlled environment with a 12 h dark/light cycle at a room temperature of 22–24 °C. Mice were divided into three experimental groups. The first group (*n*=4) was maintained on normal chow diet purchased from Zeigler Bros., Inc. (Cat. No. NIH-31M Auto, Gardners, PA, USA). The second group (*n*=5) was fed a HFD obtained from Research Diets Inc. (Cat. No. D12429, New Brunswick, NJ, USA) for 12 weeks. The third group (*n*=6) was fed a HFD together with an intraperitoneal injection of 10 mg kg^−1^ FA^28^ every other day for 12 weeks. Food intake was monitored for 3 consecutive days at 8am and 5pm on 14-week-old mice. FA was purchased from Sigma (Cat. No 128708, St. Louis, MO, USA) and dissolved in 1% Tween 80, 1% DMSO and 98% saline. The control groups (normal chow and high fat diet) were injected with vehicle.

### Glucose (GTT), pyruvate (PTT) and insulin (ITT) tolerance tests

For the GTT and PTT, mice were fasted overnight for 16–18 h and provided with water *ad libitum*. The following morning, mice were housed in individual cages and allowed to acclimatize for 2 h followed by intraperitoneal injection of 1.2 g kg^−1^ glucose (Sigma) for GTT and 2 gkg^−1^ sodium pyruvate (Sigma) for PTT. GTT was performed on 15-week-old mice; the NC (normal chow)-fed cohort (*n*=4) had a body weight of 27.6±0.3 g, the HFD-fed cohort (*n*=5) had a body weight of 41.7±1.2 g, and the HFD+FA cohort (*n*=6) had a body weight of 35.6±0.6 g. PTT was performed on 17-week-old mice; the NC-fed cohort (*n*=4) had a body weight of 29.7±0.5 g, the HFD cohort (*n*=4) had a body weight of 48.2±1.4 g and the HFD+FA (*n*=6) had a body weight of 42.4±0.8 g. For ITT, the mice were fasted and stabilized for 2 h in individual cages with free access to water. Insulin, 0.9 U kg^−1^, (Eli Lilly and Company, IN, USA) was administered intraperitoneally. ITT was performed on 16-week-old mice; the NC-fed cohort (*n*=4) had a body weight of 29.6±0.2 g, the HFD cohort (*n*=5) had a body weight of 48.9±0.6 g and the HFD+FA cohort (*n*=6) had a body weight of 40.9±0.7 g. Blood samples were obtained from a tail nick and the blood glucose levels were measured at 0, 20, 40, 60, 90, 120 and 150 min using a commercial glucometer (Bayer HealthCare, Mishawaka, IN, USA).

### Hormone measurement

Blood serum was collected at 10:00 am from 18 week old mice and the insulin and leptin levels were measured using an ELISA kit obtained from the Morinaga Institute of Biological Science (Yokohama, Japan) following the manufacturer’s protocol.

### Cell culture

The hypothalamic N1,^29^ hepatic HepG2^30^ and human embryonic kidney (HEK293) cells^10^ were cultured in Dulbecco's Modified Eagle Medium supplemented with 10% fetal bovine serum and 1% penicillin-streptomycin. The cells were maintained at 37 °C and 5% CO_2_.

### RNA isolation and real-time quantitative PCR

RNA was isolated from cells and tissue samples using Trizol (Life Technologies, Carlsbad, CA, USA), following the manufacturer’s protocol. One microgram of total RNA was used to synthesize cDNA using the high capacity cDNA reverse transcription kit (Applied Biosystems, Foster City, CA, USA). Q-PCR was performed using the SYBR Green PCR master mix from Applied Biosystems. The mouse Q-PCR primer sequences are listed below: PEPCK; 5′-CGCAAGCTGAAGAAATATGACAA-3′ and 5′-TCGATCCTGGCCACATCTC-3′, G6P; 5′-TGGGCAAAATGGCAAGGA-3′ and 5′-TCTGCCCCAGGAATCAAAAAT-3′, NPY; 5′-CTACTCCGCTCTGCGACACT-3′ and 5′-AGTGTCTCAGGGCTGGATCT-3′, AgRP; 5′-CGGCCACGAACCTCTGTAG-3′ and 5′-CTCATCCCCTGCCTTTGC-3′, POMC; 5′-CAGGTCCTGGAGTCCGAC-3′ and 5′-CATGAAGCCACCGTAACG-3′, CART; 5′-AGAAGAAGTACGGCCAAGTC-3′ and 5′-GGACAGTCACACAGCTTCC-3′. The human Q-PCR primers sequences are as follows, PEPCK; 5′-CAGGCGGCTGAAGAAGTATGA-3′ and 5′-AACCGTCTTGCTTTCGATC-3′, G6P; 5′-GCCACATCCACAGCATCTATAA-3′ and 5′-CCAGAGTCCACAGGAGGTCTAC-3′.

### Western blot

Protein was isolated from cells and tissue samples using radioimmunoprecipitation assay buffer enriched with protease and phosphatase inhibitors (Roche, Basel, Switzerland). Equal amounts of protein were loaded and separated on sodium dodecyl sulphate-acrylamide gels and then incubated with specific antibodies. The following primary antibodies were used: pAKT (4060S), AKT (2967S), pFOXO1 (9461S), FOXO1 (2880S), Lamin A/C (2032S) and PEPCK (12940S) from Cell Signaling Technology (Danvers, MA, USA). GAPDH (SC-25778) was from Santa Cruz Biotechnology (Santa Cruz, CA, USA) and G6P (ab83690) was from Abcam (Cambridge, UK). The protein levels were detected using Pierce ECL western blotting substrate (Waltham, MA, USA) following the standard western blot procedure, as described previously.^10^ Images were captured with a UVP Bio-Spectrum 600 imaging system (Ultra-Violet Products Ltd. Cambridge, UK).

### Cell fractionation

HEK 293 cells were harvested and the cell membranes lysed using ice-cold buffer A containing 10 mM 4-(2-hydroxyethyl)-1-piperazineethanesulfonic acid (pH 7.9), 10 mM KCL, 0.1 mM EDTA (pH 8.0), 0.1 mM EGTA (pH 8.0), 1 mM DTT, and protease and phosphates inhibitors. 10% Triton X-100 was added to the samples followed by 1 min centrifugation. The cytoplasmic supernatant was then collected. The pellet was then lysed with ice cold buffer C containing 20 mM 4-(2-hydroxyethyl)-1-piperazineethanesulfonic acid (pH 7.9), 400 mM NaCl, 1 mM EDTA (pH 8.0), 1 mM EGTA (pH 8.0), 1 mM DTT and protease and phosphatase inhibitors. The supernatant containing the nuclear extract was collected. Protein levels in the cytoplasmic and nuclear extracts were detected following the standard western blot procedure. GAPDH and Lamin A were used as internal controls for the cytoplasmic and nuclear extracts, respectively.

### FoxO1 localization assay

FoxO1 localization was assayed by transfecting HEK 293 cells with FoxO1-GFP using Lipofectamine 2000 (Thermo Fisher Scientific, Waltham, MA, USA), followed by 6 h FA treatment. The cells were fixed in 4% paraformaldehyde for 10 min and washed three times with phosphate-buffered salinePBS. Slides were mounted using VECTASHIELD mounting medium with DAPI (Vector Laboratories, Inc. Burlingame, CA, USA) and visualized using the Olympus BX51 fluorescence microscope (Olympus Corporation, Tokyo, Japan).

### Luciferase assay

PEPCK and G6P luciferase constructs were kindly provided by Dr Hueng-Sik Choi, as previously described.^31^ AgRP, NPY and POMC luciferase constructs were kindly provided by Dr Min-Seon Kim, as previously described.^19^ HEK 293 cells were transfected with specific DNAs using Lipofectamine 2000 (Thermo Fisher Scientific) following the manufacturer’s protocol, and renilla (100 ng) was co-transfected as an internal control. Cells were lysed using luciferase lysis buffer composed of 25 mM Tris phosphate pH 7.8, 2 mM DTT, 2 mM EDTA, 10% glycerol and 1% Triton X-100. The cell lysate samples were assayed in triplicate after injection of luciferase and renilla substrates using the BioTek Synergy TM 2 machine (BioTek Instruments Inc., Winooski, VT, USA). The luciferase substrate (pH 8.0) was composed of 200 mM Tris-HCL, 15 mM MgSO_4_, 0.1 mM EDTA, 25 mM DTT, 1 mM ATP, 0.2 mM coenzyme A and 200 μM luciferin. The renilla substrate (pH 5.0) was composed of 1.1 M NaCl, 2.2 mM Na_2_EDTA, 0.22 M KH_2_PO_4_ (pH 5.1), 0.44 mg ml^−1^ BSA, 1.3 mM sodium azide, 1.43 μM coelenterazine.^32^ The relative luciferase activity of the FA treatment was calculated as a ratio of luciferase and renilla activity normalized to vehicle treatment.

### Histological analysis

White adipose tissue and liver samples were fixed in 4% neutral buffered formalin. The tissue samples were then paraffin embedded and cut into 5 μm slices. The slices were stained with hematoxylin and eosin (H&E) following the standard H&E procedure. The stained slides were visualized by a Nikon Digital Camera DXM1200 microscope system (Nikon Corporation, Tokyo, Japan).

### Statistical analysis

The data were analyzed using GraphPad Prism 5.0 software. Statistical comparisons were performed using Student’s *t*-test or ANOVA, and results with a *P*-value <0.05 were considered to be statistically significant. All results are expressed as the mean±s.e.m. as indicated in the figure legends.

## Results

### FA improves metabolic phenotype in HFD-induced obese mice

**Figure 1:**
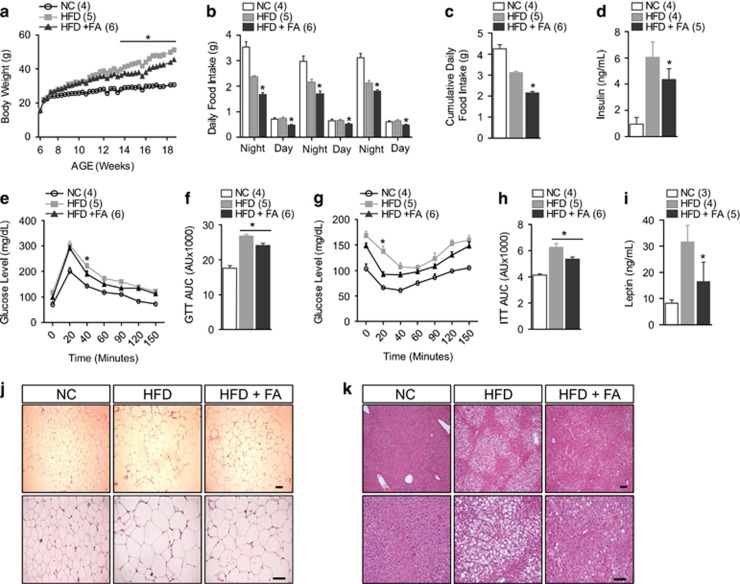
Improved metabolic profile after FA treatment. Weekly body weight (**a**), day and night food intake (**b**), cumulative daily food intake (**c**), and blood insulin level (**d**) in mice fed NC, HFD or HFD+FA. Note that day and night food intake was measured at 8am and 5pm for 3 consecutive days. Glucose tolerance test (GTT) (**e**) and area under curve (AUC) for the GTT (**f**). Insulin tolerance test (ITT) (**g**) and AUC for the ITT (**h**). Plasma leptin level (**i**). Representative images for H&E staining in white adipose tissue (**j**) and liver (**k**). The lower panel indicates a higher magnification from the upper panel. The number of animals is expressed in parenthesis. The values are mean±s.e.m. (*P<0.05, Student’s *t*-test, two-way ANOVA for **a**, **e** and **g**). NC, normal chow. HFD, high-fat diet. FA, 4-hydroxy-3-methoxycinnamic acid (ferulic acid). Scale bar, 100 μm.

To investigate the metabolic effects of FA, male BL6 mice were divided into three groups. One group was maintained on normal chow diet, another group on HFD and the third group was fed HFD plus a 10 mg kg^−1^ FA intraperitoneal injection every other day for 12 weeks. The FA-treated mice showed significantly lower body weight gain under HFD compared with vehicle treated littermates (Figure 1a), indicating that FA might have protective effects against HFD-induced obesity. Next, we monitored food consumption to examine whether the decrease in body weight gain observed in FA-treated mice resulted from altered food intake. Intriguingly, FA treated mice exhibited a significant decrease in day and night food consumption as well as cumulative daily food intake (Figure 1b and c). Together with the regulation of body weight, assessment of serum insulin levels showed that the HFD-induced increase in serum insulin level was significantly blunted by FA treatment, suggesting that FA might improve glucose and insulin sensitivity (Figure 1d). To further investigate the effect of FA on glucose and insulin homeostasis, we performed GTT and ITT. The FA-treated mice exhibited improved glucose tolerance and increased insulin sensitivity compared with control littermates (Figure 1e–h). In general, obesity is associated with increased leptin levels in the blood as well as the development of leptin resistance. HFD has also been shown to contribute to increased plasma leptin levels.^33, 34, 35^ In this study, we observed that FA markedly reduced the HFD-induced increase in serum leptin level (Figure 1i) and decreased food intake (Figure 1b and c), suggesting that FA might be involved in the regulation of leptin sensitivity. This improved metabolic phenotype in the FA-treated mice was associated with a marked reduction in the size and accumulation of lipid droplets in the white adipose tissue and liver compared with the HFD control group (Figure 1j and k). Collectively, these data highlight the beneficial metabolic effects of FA in the protection against HFD-induced obesity.

### Suppression of HFD-induced hepatic glucose production by FA

**Figure 2:**
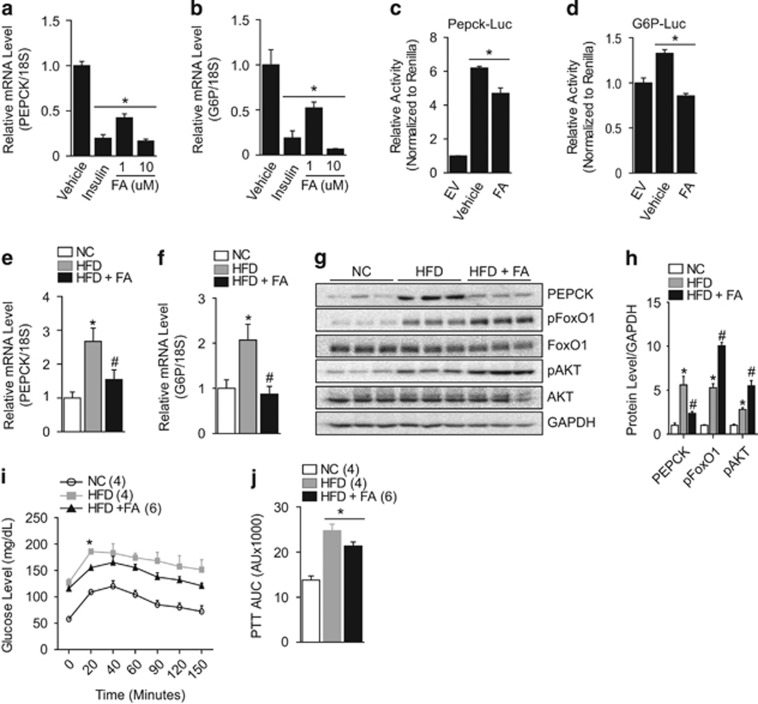
Effect of FA on the expression of hepatic gluconeogenic genes. Effect of FA on expression of PEPCK (**a**) and G6P (**b**) in HepG2 cells. Effect of FA on luciferase activity of PEPCK (**c**) and G6P (**d**) in HEK 293 cells. Inhibition of HFD-induced hepatic gluconeogenic PEPCK (**e**) or G6P (**f**) by FA treatment. Change in liver PEPCK, pAKT, and pFoxO1 after FA treatment (**g**). Densitometry of the proteins from **g** (**h**). Pyruvate tolerance test (PTT) (**i**) and AUC for PTT (**j**). The values are mean±s.e.m. (*, ^#^*P*<0.05, Student’s *t*-test, two-way ANOVA for **i**). All *in vitro* experiments were performed in triplicate. PEPCK, phosphoenolpyruvate carboxylase. G6P, glucose-6-phosphatase.

Several studies have suggested that improved glucose and insulin tolerance is associated with the inhibition of hepatic glucose production.^36, 37, 38^ These, together with the improved glucose, insulin, and leptin levels in the FA-treated group, led us to hypothesize that FA might reduce hepatic glucose production in HFD-induced obese mice. To address this question, we examined the expression of the rate limiting enzymes for gluconeogenesis, PEPCK and G6P. The expression of PEPCK and G6P was significantly blunted by FA treatment and this suppression was comparable with insulin treatment in HepG2 cells (Figure 2a and b). Luciferase assays also showed a marked reduction in PEPCK and G6P promoter activity after FA treatment (Figure 2c and d). Consistent with the *in vitro* results, FA markedly blunted the HFD-induced PEPCK and G6P increase observed in the liver (Figure 2e–h). Finally, we performed PTT to directly assess the role of FA in hepatic glucose homeostasis by challenging mice with the gluconeogenic precursor, pyruvate. As shown in Figure 2i and j, the FA treated mice showed significantly improved pyruvate tolerance as evidenced by a significant reduction in area under the curve compared with vehicle-treated HFD-obese littermates. Taken together, our data suggest that FA improves insulin sensitivity by reducing hepatic glucose output, possibly by inhibiting the expression of gluconeogenic genes.

### Inactivation of FoxO1 is required for the suppression of hepatic gluconeogenic genes by FA

**Figure 3:**
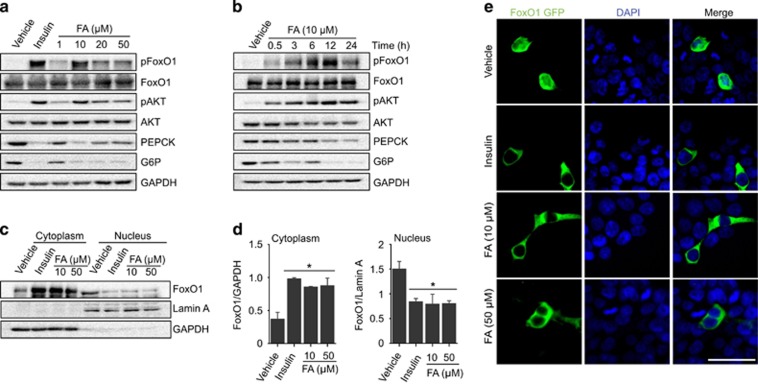
Inhibition of FoxO1 by FA through phosphorylation and cytoplasmic localization. Dose-dependent effect of FA on pAKT, pFoxO1, PEPCK and G6P (**a**). Note that 100 nM insulin was used as a positive control for the phosphorylation of FoxO1. Time-dependent effect of FA on pAKT, pFoxO1, PEPCK and G6P in HepG2 cells (**b**). Cytoplasmic or nuclear localization of FoxO1 after FA treatment (**c**). Quantification of FoxO1 protein levels in the cytoplasm and nucleus (**d**). Fluorescence imaging of FoxO1-GFP after FA treatment (**e**). GAPDH and Lamin A were used as fractionation markers for the cytoplasm and nucleus, respectively. The values are mean±s.e.m. (*P<0.05, Student’s *t*-test and one-way ANOVA). Scale bar, 50 μm.

FoxO1 is a transcription factor localized in the nucleus in its active state. However, upon phosphorylation, mainly by AKT, it is translocated to the cytoplasm where it is inactivated and eventually degraded. FoxO1 is known to regulate the expression of PEPCK and G6P in the liver to maintain glucose homeostasis.^39^ Given that FA treatment significantly increased the phosphorylation of both FoxO1 and AKT accompanied by suppression of the HFD-induced expression of PEPCK and G6P in the liver, we hypothesized that FA exerted its suppressive effects on PEPCK and G6P by modulating the activity of FoxO1 (Figure 2e–h). To further investigate this, we administered FA dose and time dependently and monitored FoxO1 activity in HepG2 cells. FA treatment induced a significant phosphorylation of AKT and FoxO1 dose and time dependently, with the highest effect observed by 10 μM FA treatment (Figure 3a and b and Supplementary Figure 1a and b). In addition, FA significantly suppressed PEPCK and G6P protein levels in a dose- and time-dependent manner (Figure 3a and b and Supplementary Figure 1c and d). The phosphorylation of FoxO1 by FA facilitated FoxO1 nuclear exclusion in a manner comparable to insulin-induced FoxO1 translocation (Figure 3c–e). Taken together, these data show that FA might facilitate the phosphorylation and inactivation of FoxO1 and thereby suppress the expression of the FoxO1 gluconeogenic targets, PEPCK and G6P.

### FA inhibits hypothalamic orexigenic neuropeptides by FoxO1 inactivation

**Figure 4:**
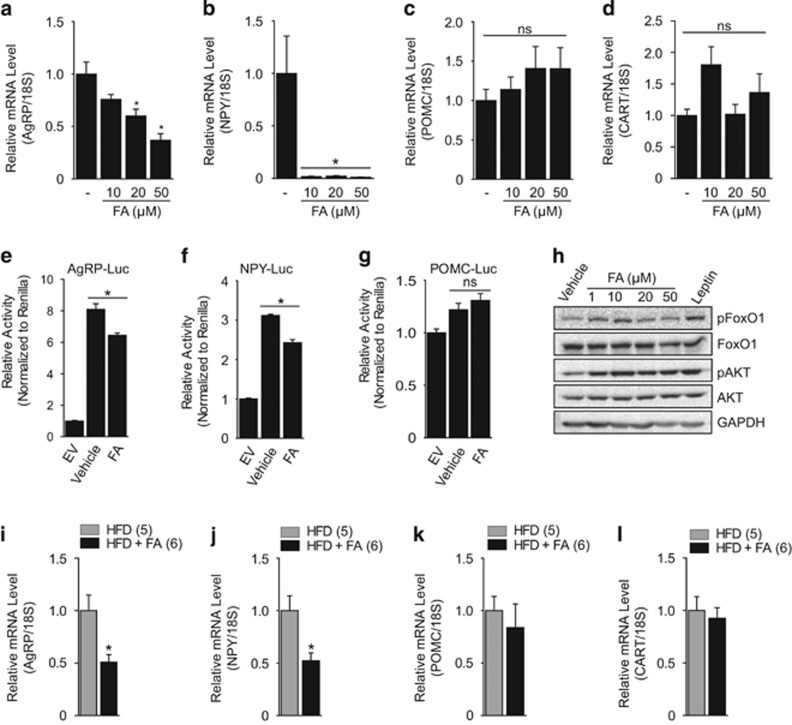
FA suppresses the expression of hypothalamic orexigenic neuropeptides. Effect of FA on the expression of AgRP (**a**), NPY (**b**), POMC (**c**) and CART (**d**) in hypothalamic N1 cells. Effect of FA on the luciferase activity of AgRP (**e**), NPY (**f**) and POMC (**g**) in HEK 293 cells. Dose-dependent effect of FA on pFoxO1 and pAKT in hypothalamic N1 cells (**h**). Note that leptin (100 nM) was used as a positive activator of pFoxO1 in N1 cells. Effect of FA on the expression of AgRP (**i**), NPY (**j**), POMC (**k**) and CART (**l**) in the hypothalamus of mice fed HFD or HFD+FA. The number of animals is expressed in parenthesis. All *in vitro* experiments were performed in triplicate. The values are mean±s.e.m. (*p<0.05, Student’s *t*-test and one-way ANOVA). EV, empty vector. ns, not significant.

Another metabolic characteristic observed in the FA-treated mice was a reduction in calorie intake compared with vehicle-treated control mice (Figure 1a–c). It is well known that calorie intake is tightly regulated by a central feeding system in response to satiety signals generated by the peripheral system. Following a study reporting that FA is capable of crossing the blood–brain barrier,^40^ we hypothesized that FA might be involved in the modulation of the hypothalamic neuropeptides that regulate food consumption. Two distinct neuronal populations that regulate feeding behavior, the orexigenic NPY/AgRP neurons and the anorexigenic POMC/CART neurons, are expressed in the arcuate nucleus of the hypothalamus. Therefore, we examined the effect of FA on the expression of orexigenic and anorexigenic genes using the N1 hypothalamic cell line.^41^ Surprisingly, FA treatment significantly reduced the expression of AgRP and NPY (Figure 4a and b), with no significant change in the expression of POMC and CART (Figure 4c and d). Next, we examined the promoter activity of AgRP, NPY, and POMC via luciferase assay after FA treatment. Although there was no observable effect on POMC, FA markedly reduced the transcriptional activity of AgRP and NPY (Figure 4e–g). It has been reported that FoxO1 stimulates the expression of the orexigenic neuropeptides AgRP and NPY in the hypothalamus and that phosphorylation of FoxO1 promotes its nuclear export and hence inhibits the expression of orexigenic neuropeptides.^18, 19, 42^ Having observed that FoxO1 was involved in the regulation of gluconeogenic genes in FA-treated mice (Figures 2 and 3), we investigated the dose-dependent effect of FA on FoxO1 activity in hypothalamic cells. As shown in Figure 4h, FA significantly inhibited FoxO1. To further confirm the effect of FA on the expression of orexigenic and anorexigenic neuropeptides, we isolated hypothalamic RNA from vehicle and FA-treated mice and assayed gene expression using Q-PCR. The FA treated mice showed a significant reduction in both AgRP and NYP (Figure 4i and j) with no observable change in POMC and CART (Figure 4k and l). These results suggest that FA might suppress food consumption by regulating the expression and activity of orexigenic neuropeptides in the hypothalamus through inactivation of FoxO1.

## Discussion

Phytochemicals are natural compounds derived from plant and marine algae extracts that have emerged as important nutraceuticals and supplementary treatments in the fight against obesity and type II diabetes.^30, 43, 44^ In this study, we explored the molecular mechanisms mediating the beneficial metabolic effects of FA using a HFD-induced obese mouse model and *in vitro* analyses. We report that FA suppressed energy consumption by inhibiting the expression and transcriptional activity of orexigenic genes. FA also contributed to improved insulin sensitivity by suppressing hepatic glucose production. Further, we show that this FA-induced reduction in food intake and suppression of hepatic glucose production was accompanied by an overall improvement in whole-body energy homeostasis characterized by increased insulin sensitivity, glucose tolerance, and reduced fat droplet accumulation in the liver, especially under the HFD condition. Treatment with FA inactivated the transcription factor FoxO1, which is known to regulate the expression of orexigenic peptides in the hypothalamus and gluconeogenic genes in the liver. We therefore suggest inactivation of FoxO1 by FA treatment as a common molecular mechanism through which FA exerts its beneficial metabolic effects by suppressing the expression of orexigenic and gluconeogenic genes. Further, it would be interesting to investigate whether FA also exerts beneficial metabolic effect in normal chow-fed animals by regulating similar mechanisms observed in HFD condition.

Obesity occurs as a result of an imbalance between energy consumption and energy expenditure.^45^ In the current study, the FA-treated mice showed resistance to HFD-induced weight gain associated with reduced food intake compared with the vehicle-treated littermates (Figure 1a–c). As the hypothalamus has an important role in regulating energy intake,^3, 4^ we investigated whether FA is involved in the regulation of feeding behavior in the hypothalamus. Interestingly, our results show that FA suppressed orexigenic NPY and AgRP expression with no significant effect on the anorexigenic peptides POMC and CART. The orexigenic and anorexigenic neurons regulate feeding behavior by integrating peripheral and central stimuli, including neurotransmitters and hormones. For example, the satiety hormone leptin is known to exert its anorexigenic effects by stimulating POMC neurons and inhibiting AgRP and NPY neurons, whereas ghrelin, an orexigenic hormone produced in the gut, is known to suppress POMC neurons and stimulate AgRP and NPY neurons.^46^ Whereas the suppression of orexigenic neurons is not always accompanied by the activation of anorexigenic neurons, and vice versa, it would be interesting to further investigate why the FA-induced suppression of orexigenic genes was not accompanied by a significant change in anorexigenic genes by focusing on the effect of FA on energy expenditure and the expression of the melanocortin receptors that mediate the anorexigenic effects of POMC. In addition, previous reports have indicated that suppression of AgRP and NPY is accompanied by the activation of the sympathetic tone important for energy expenditure regulation.^47, 48, 49, 50^ Therefore, future studies should examine whether FA has a role in the regulation of the sympathetic nervous system or energy expenditure.

Excessive hepatic glucose production has been associated with obesity as well as the development of type II diabetes.^36^ In this study, we found that FA markedly blunted the HFD-induced increase in PEPCK and G6P and suppressed hepatic glucose production, leading to improved hepatic glucose homeostasis (Figure 2). In addition, GTT and ITT tests showed that FA improved glucose tolerance and insulin sensitivity under HFD condition and that this was accompanied by a significant reduction in serum insulin and leptin levels (Figure 1). The expression of the gluconeogenic genes PEPCK and G6P is tightly regulated by the transcription factor FoxO1; functional inhibition of FoxO1 was reported to reduce hepatic gluconeogenic activity.^51, 52, 53^ We found that FA treatment strongly induced phosphorylation of FoxO1 and that this phosphorylation led to the nuclear exclusion and subsequent reduced activity of FoxO1 on PEPCK and G6P, resulting in improved hepatic glucose homeostasis by decreasing HFD-induced hepatic glucose production. In addition to FoxO1, it has been shown that cAMP response element binding (CREB) is a critical factor that modulates hepatic glucose production through direct regulation of PEPCK and G6P.^54^ The transcriptional activity of FoxO1 and CREB is modulated by transcriptional co-activators, including CREB-binding protein/p300, CREB-regulated transcription co-activator 2, and peroxisome proliferator-activated receptor gamma co-activator 1 alpha.^54^ Therefore, whether FA has a role in the regulation of CREB-mediated hepatic glucose homeostasis or facilitates the interaction between FoxO1 and CREB, with the associated transcriptional co-activators remains to be investigated.

In this study, we observed the phosphorylation and nuclear exclusion of FoxO1 by FA. FoxO1 is regulated by various factors that modulate its subcellular localization, DNA-binding, transcriptional activity and even its protein expression levels. This is achieved mainly through post-translational modifications, including phosphorylation, acetylation and ubiquitination. The serine/threonine kinase AKT is one of the major kinases known to phosphorylate and facilitate the cytoplasmic localization of FoxO1.^55^ Our studies show that FA induced the phosphorylation of AKT with the subsequent phosphorylation of FoxO1. Although AKT seems to be primarily involved in phosphorylation of FoxO1, we currently do not know the exact upstream kinases that led to FA-induced FoxO1 phosphorylation. Therefore, it would be interesting to conduct further studies to elucidate the mechanism by which FA induces the phosphorylation of FoxO1. As leptin and insulin regulate energy homeostasis by converging at the PI3K/AKT/FoxO1 pathway, it would also be of great interest to examine whether FA has potentiating or synergist effects on the metabolic activities of insulin and leptin.

In conclusion, our study corroborates the beneficial metabolic effects of FA and highlights the role of FA as an alternative therapeutic agent in the fight against metabolic syndrome. We indicate that FA might exert its beneficial metabolic effects in the hypothalamus as well as in the periphery by regulating energy intake and hepatic glucose homeostasis. Further, our data suggest that FA suppresses the expression of orexigenic genes and hepatic gluconeogenic genes by phosphorylating and inhibiting FoxO1.
