# Supplementary material for: 4-hydroxy-3-methoxycinnamic acid regulates orexigenic peptides and hepatic glucose homeostasis through phosphorylation of FoxO1
Source: Exp Mol Med. 2018 Feb 2;50(2):e437–. doi: 10.1038/emm.2017.253 (PMC5903816; doi:10.1038/emm.2017.253)
Supplement: Supplementary Figure 1 [file emm2017253x1.pdf]

Sup. Fig. 1

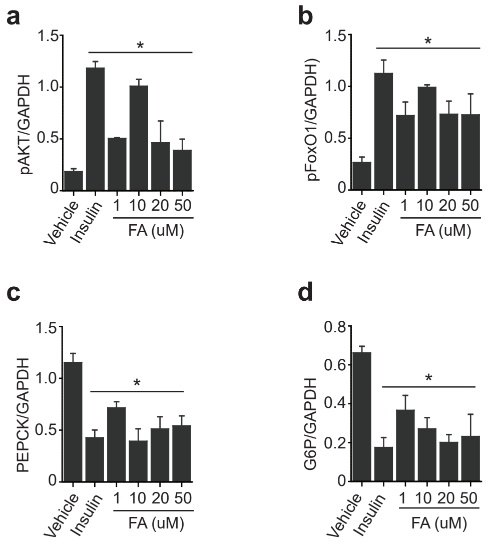

Supplementary Figure 1. Dose-dependent effect of FA on pAKT, pFoxO1, PEPCK, and G6P levels.

Dose-dependent effect of FA treatment on the phosphorylation of AKT (a) and FoxO1 (b), and on the levels of PEPCK (c) and G6P (d). The values are mean ± SEM (*$p < 0.05$, Student's *t*-test and one-way ANOVA).
